# Effectiveness of a combined strategy to improve therapeutic compliance and degree of control among patients with hypercholesterolaemia: a randomised clinical trial

**DOI:** 10.1186/1471-2261-15-8

**Published:** 2015-01-19

**Authors:** Ignacio Párraga-Martínez, Joseba Rabanales-Sotos, Fernando Lago-Deibe, Juan M Téllez-Lapeira, Francisco Escobar-Rabadán, Alejandro Villena-Ferrer, Mariano Blasco-Valle, José M Ferreras-Amez, Susana Morena-Rayo, José M del Campo-del Campo, Maria Candelaria Ayuso-Raya, José J Pérez-Pascual

**Affiliations:** La Roda Health Centre, Health Care Service of Castilla-La Mancha, C/Mártires 63, 02630 Albacete, La Roda Spain; Cuenca Faculty of Nursing, University of Castilla-La Mancha, C/Camino de Pozuelo s/n, 16071 Cuenca, Spain; Sárdoma Health Centre, Health Care Service of Galicia, C/Baixada a Laxe 76, 36214 Vigo, Spain; Albacete Area Vb Health Centre, Health Care Service of Castilla-La Mancha, C/Profesor Macedonio Jiménez s/n, 02006 Albacete, Spain; Albacete Area IV Health Centre, Health Care Service of Castilla-La Mancha, C/Seminario 4, 02006 Albacete, Spain; San Clemente Health Centre, Health Care Service of Castilla-La Mancha, C/Ancha s/n, 16600 Cuenca, San Clemente, Spain; Delicias Sur Health Centre, Health Care Service of Aragón, C/Manuel Dronda 1, 50009 Zaragoza, Spain; Royo Villanova Hospital, Health Care Service of Aragón, C/San Gregorio 30, 50015 Zaragoza, Spain; Hellín 2 Health Centre, Health Care Service of Castilla-La Mancha, C/Turbas de Cuenca 15, 02400 Albacete, Hellín Spain; Almansa Health Centre, Health Care Service of Castilla-La Mancha, C/San Juan s/n, 02640 Albacete, Almansa Spain

**Keywords:** Hypercholesterolaemia, Medication adherence, Primary health care

## Abstract

**Background:**

In subjects with hypercholesterolaemia, cholesterol values remain above guideline levels. One of the limiting factors to the achievement of goals in such patients is therapeutic non-adherence. The aim of this study is to assess the effectiveness of an intervention designed to improve control of hypercholesterolaemic patients, consisting of a combined strategy that would include the delivery of printed information, treatment-compliance check cards and the dispatch of text messages as complementary measures in support of the intervention at the general practitioner’s practice.

**Methods/Design:**

A randomised, parallel-group clinical trial will be conducted at the family medicine outpatient facilities of eight health centres in three of Spain’s Autonomous Regions (Comunidades Autónomas), covering a total of 358 subjects aged 18 years or over with diagnosis of hypercholesterolaemia. Patients in the intervention group will be supplied with printed material with information on the disease and its management, mobile-telephone text messages with guideline summaries, reminders of forthcoming appointments and/or arrangements for making new appointments in the event of non-attendance, and self-report cards to check compliance with recommendations. Both groups -intervention and control- will receive routine recommendations from their physicians in accordance with current European clinical practice guidelines for hypercholesterolaemia and cardiovascular risk management. As regards the measurements to be made, the main variable is the proportion of subjects who attain the low density lipoprotein cholesterol levels set as a target across a follow-up period of 24 months. The secondary variables are as follows: adherence to recommendations on lifestyle and adherence to drug treatment; variation in lipid profiles and cardiovascular risk levels; appearance of cardiovascular events; physical activity; food consumption; smoking habit; anthropometric measures; blood pressure; health problems; use of hypolipidaemic agents; socio-demographic data; beliefs and expectations about preventive recommendations; and degree of satisfaction with the combined strategy.

**Discussion:**

Should this intervention prove effective, a recommendation could be issued on the application of this combined strategy to subjects with hypercholesterolaemia. It is a simple, relatively inexpensive intervention.

**Trial registration:**

ClinicalTrials.gov: NCT02314663.

## Background

Hypercholesterolaemia is one of the main cardiovascular disease risk factors (CDRFs) and, according to different studies undertaken in Spain, its prevalence in terms of total plasma cholesterol concentrations of over 250 mg/dl, extends to 20%-23% of the adult population [1, 2].

The prevention and treatment of dyslipidaemia should take the remaining CDRFs into account, with its management being based on the calculation of cardiovascular risk. While most European clinical practice guidelines use a Framingham or Systematic Coronary Risk Evaluation (SCORE) function chart [3] for this purpose, the Registre Gironí del Cor (REGICOR) study [4] offers an alternative, using a series of tables adapted and validated in Spain. Management of dyslipidaemia includes both pharmacological and non-pharmacological measures (diet and physical activity), with the latter being recommended for 3 to 6 months in primary prevention prior to the introduction of hypolipidaemic treatment [5–7].

Furthermore, the degree of control of CVR (cardiovascular risk) factors is low, both in primary and in secondary prevention. Despite guidelines and new hypolipidaemic agents, the results of different studies nonetheless show that cholesterol values remain above guideline targets [8]. Studies, such as HISPALIPID [9] and PREVENCAT [10], have revealed poor control of subjects with dyslipidaemia, with the designated targets being attained in approximately 32% of cases. The EUROASPIRE II study reported that 64% of patients with coronary disease took hypolipidaemic agents, and that approximately 53% failed to display adequate therapeutic control [8]. Similar results as regards achievement of goals were reported by the international EUROASPIRE III [11] and LTAP-2 [12] studies, which included data on Spain. Among the possible causes of this situation are factors linked to hypolipidaemic agents, adherence to prescribed treatment and physician-led prescription strategy.

Bearing in mind that over half of all patients with coronary disease and over two thirds of subjects judged to be at high cardiovascular risk fail to attain target lipid values [13], it would seem necessary to implement strategies that enhance adherence to lifestyle changes and compliance with drug therapy. There is evidence of and wide consensus about the benefits of the diet, exercise and drugs to be used, both in primary prevention and in secondary prevention in particular, for the purpose of achieving good control of patients with hypercholesterolaemia.

Among the factors related to adherence to lifestyles and drug therapy in dyslipidaemia, it should be noted that the asymptomatic nature of this disease may contribute to the poor results obtained [7]. Other possible factors are an absence of perception of a risk to health and negligible participation in decision-making [14].

One of the limiting factors in the achievement of targets in hypercholesterolaemic patients is therapeutic non-adherence, which decreases across time [15, 16]. Moreover, such non-adherence should not be taken to refer to medications exclusively, but also to the remaining guidelines. In 2003 the World Health Organization (WHO) defined the term adherence as, “The extent to which a person’s behaviour - taking medication, following a diet, and/or executing lifestyle changes, corresponds with agreed recommendations from a health care provider” [17]. Among dyslipedaemic patients the percentage of non-compliers ranges from 22% to 57% [18], with different studies observing that, in over half of all cases, this is due to forgetfulness when it comes to taking the medication [19]. Other studies have similarly reported a lack of compliance with lifestyle recommendations [20].

Various strategies have been used to improve compliance and, by extension, control of patients. The results of the study conducted by Márquez et al. [21] into the effectiveness of an isolated telephone intervention on compliance with drug treatment, indicated better adherence, yet the patients nevertheless displayed poor control of their dyslipidaemia. This may indicate that improvement in pharmacological adherence alone is not enough to attain designated targets, and that to achieve adequate control, it is essential to follow recommendations other than those which are purely pharmacological. At present, evidence is not being transferred to clinical practice, either here in Spain or in the rest of Europe, and targets are thus not being achieved.

In addition, the strategies that have most improved compliance among subjects with other risk factors, such as hypertension, do not include specific activities, such as pamphlets or telephone alerts, but rather combined activities, such as group sessions coupled with mail-based back-up and support [22], or alternatively, the delivery of printed matter, appointment-reminder telephone calls and mail with educational messages about the disease [23]. In this respect, there would seem to be agreement on the need to use a combination of strategies to improve adherence, thus surpassing the possible benefits of single strategies implemented one at a time [24, 25]. Hence, studies that have used printed information in tandem with individual health education [26, 27] have displayed better results than those that have evaluated just one of these interventions [28, 29], and have successfully managed to increase knowledge about dyslipidaemia and its management. Likewise, strategies exclusively targeted at recording the taking of medication, e.g., telephone calls in the case of dyslipidaemias, have achieved little improvement in compliance [30], with results showing improvement when different reminder combinations have been used [31].

The choice of one method or another should also depend on the population to be targeted and, when it comes to choosing an intervention, consideration should be given to the possible risk entailed in only implementing specific actions that have no continuity and are excessively costly (in terms of time or money) for the expected benefit [32].

In the light of the above, we therefore propose a combined strategy to improve compliance with the recommendations contained in clinical practice guidelines for management of dyslipidaemia, both as to lifestyles (diet, physical activity, alcohol and loss of weight) and as to hypolipidaemic agents. It is a strategy targeted at improving knowledge of dyslipidaemia, increasing compliance with recommendations and facilitating greater patient participation. To this end, a controlled study should be undertaken to examine the effectiveness of such a strategy in terms of adherence to recommendations and control of the cholesterol values of subjects with hypercholesterolaemia.

The proposed study follows the recommendations made by Haynes [33] for studies that assess compliance strategies, inasmuch as it investigates an easily applicable practical intervention, uses a randomised-clinical-trial design, and envisages measuring both therapeutic adherence and the degree of lipid control achieved.

### Study objectives

The main aim of this study is to assess the effectiveness of an intervention designed to improve control of hypercholesterolaemic patients, consisting of a combined strategy that would include the delivery of printed information, text messages and treatment-compliance check cards as complementary measures in support of the intervention at the general practitioner’s (GP) practice.

The secondary objectives would be: 1) to ascertain whether, with respect to routine GP care, the combined intervention strategy improves therapeutic adherence, both pharmacological and non-pharmacological, of hypercholesterolaemic patients, across a follow-up period of 24 months; 2) to ascertain whether, with respect to routine GP care, the combined intervention strategy increases the proportion of patients who attain the low density lipoprotein cholesterol (LDL-C) values set for adequate control, across a follow-up period of 24 months; 3) to quantify the effect of the combined intervention strategy on the remaining lipid-profile-defining parameters, across follow-up; 4) to quantify the effect of the combined strategy on the reduction of cardiovascular risk in patients with hypercholesterolaemia, across follow-up; 5) to identify the factors that contribute to improving the effectiveness of the combined intervention strategy on reducing lipid values, not only in relation to patients’ health status but also in relation to their socio-demographic variables; and, 6) to assess the acceptance and degree of satisfaction of the hypercholesterolaemic patients undergoing the intervention.

## Methods/Design

### Design

Randomised, parallel-group clinical trial in which a group of hypercholesterolaemic patients, undergoing a combined intervention targeted at improving treatment adherence and degree of lipid control as complementary measures in support of the intervention at the GP’s practice, will be compared to another group of hypercholesterolaemic patients who will solely receive routine GP care.

### Study location and subjects

Participants will be recruited from among patients attending family medicine outpatient facilities at 8 health centres situated in three health areas of three of Spain’s Autonomous Regions (Comunidades Autónomas), namely, Castile-La Mancha (Albacete Health Area), Aragon (Zaragoza Health Area) and Galicia (Vigo Health Area). The target population is made up of all persons aged 18 years or over with diagnosis of hypercholesterolaemia, with the population eligible for study comprising hypercholesterolaemic subjects of this age drawn from the eight health centres in the above three participating health areas.

The study inclusion criteria require that candidates: a) be diagnosed with hypercholesterolaemia, as defined according to the criteria stipulated in the cardiovascular prevention guidelines of the 2009 Prevention Activities and Promotion of Health Programme (total cholesterol of 250 mg/dl or higher); and, b) be patients aged 18 years or over attending any of the participating health centres. The following individuals will be excluded: a) any person hindered from participating in the follow-up of the proposed intervention, e.g., illiterate subjects and non-users of mobile telephones; b) any person with a physical disability or impairment which prevents him/her from attending the follow-up visits; and, c) any person suffering from a significant chronic organic or psychiatric disease.

### Sample size

The sample size needed for analysis will be calculated using a two-sided test, based a clinically relevant difference of 20% in the proportion of subjects who attain the lipid control targets, i.e., 55% in the control group, according to data furnished by previous studies (Kotseva et al. [8, 11]; Bueno et al. [18]), and 75% in the group undergoing the proposed intervention. Assuming a statistical power of 90% and an α error of 5%, the estimated sample size would then be 155 subjects in each group (total of 310 subjects). Assuming an expected proportion of losses of 15%, the final size of each group would be 179 subjects (an overall total of 358).

### Selection of subjects

Eight health centres in the Albacete, Zaragoza and Vigo Health Areas have been chosen for strategic reasons. To prevent contamination bias, the control group participants will belong to areas different to those of the experimental groups. Accordingly, participants will be randomised on a health-area basis, with subjects then being consecutively selected.

### Intervention

Participants in the intervention group will be supplied with: a) printed matter containing information on the disease and its management (this will be handed out at each of the follow-up visits); b) mobile-telephone text messages containing guideline summaries, reminders of forthcoming appointments and/or arrangements for making new appointments in the event of non-attendance (in the periods between visits); and, c) self-report cards to check compliance with recommendations (across the entire follow-up).

Both groups, intervention and control, will receive routine recommendations from their GPs, in accordance with current European clinical practice guidelines on the management of hypercholesterolaemia and cardiovascular risk (European Guidelines on Cardiovascular Disease Prevention in Clinical Practice: Fifth Joint Task Force of the European Society of Cardiology and other societies and Guidelines for Management of Dyslipidaemias: the Task Force for the Management of Dyslipidaemias of the European Society of Cardiology and the European Atherosclerosis Society).

### Subject follow-up

Subjects will be monitored for a period of two years.

Selection will be made at primary care outpatient facilities, inviting patients who meet the inclusion criteria to participate in the study. Once they have given their consent, such patients will then be given an appointment for an initial visit and analysis.

During the initial visit, patients will be randomised to one of the two groups. Data will be collected from all participants covering medical history (socio-demographic variables, personal and family background, lifestyle habits), physical examination (anthropometric parameters, blood pressure), analysis (lipid profile, glucose) and calculation of cardiovascular risk. The routine standard recommendations, pharmacological and non-pharmacological, will be made for their treatment. An appointment will be made for the next visit.

Participants included in the intervention group will be given printed matter describing the disease and its management, along with treatment-compliance check cards, and they will be informed about the frequency and content of the mobile-telephone text messages that they will receive. Disease-management reminders will be dispatched every 15 days and reminders about attendance at pending appointments or missed appointments will be sent according to the follow-up dates.

With respect to follow-up, after the initial visit, 5 more will be made: these will take place at 2, 6, 12, 18, and 24 months. At each of these visits, anthropometric parameters (weight, BMI and waist circumference), blood pressure figures, lipid profile (total cholesterol, LDL-C, HDL-C and triglycerides), glucose and clinical history data will be recorded, and cardiovascular risk will be calculated. Data evaluating compliance with or adherence to treatment guidelines will be obtained for both groups of patients. Data on satisfaction will solely be recorded during the final visit, and then only for subjects in the intervention group.

Any of the following circumstances will be grounds for termination of the study in the case of any given participant: completion of the observation period (2 years); violation of the protocol; intercurrent disease that renders continuation of the intervention impossible; and withdrawal from the study/withdrawal of consent.

### Definitions of the primary variables and measuring methods

The main study variable is the proportion of subjects who attain the LDL-C levels set as targets by the Guidelines for Management of Dyslipidaemias and CVR, across a follow-up period of 24 months. The plasma values taken as cardiovascular prevention targets are: a) LDL-C <100 mg/dl for patients without established cardiovascular disease or diabetes mellitus; and, b) LDL-C <70 mg/dl for patients with diabetes mellitus or established cardiovascular disease.

The secondary variables are as follows:

- adherence to lifestyle guidelines and adherence to drug treatment, as seen from self-reported adherence (adapted Haynes-Sackett test), validated questionnaire (Morisky-Green test) and Likert scale with 5 response options;
- variation in plasma lipid profile levels at 2, 6, 12, 18 and 24 months (total cholesterol, LDL-C, HDL-C and triglycerides);
- variation in cardiovascular risk level across follow-up, with assessment to be performed using the SCORE (low CVR countries) and REGICOR tables;
- appearance of cardiovascular events in the observation period, i.e., ischaemic heart disease, atherothrombotic cerebrovascular disease, heart failure and peripheral arterial disease;
- physical activity, i.e., determination of the degree of aerobic physical exercise performed (active, partially active or inactive);
- determination of the frequency of food consumption;
- smoking habit, with a smoker being defined as anyone who answers affirmatively to the question, “Do you smoke?”;
- anthropometric measures, i.e., weight, height, body mass index (BMI), and waist circumference;
- systolic and diastolic blood pressure (two measurements), with the result being the mean of the two results;
- health problems (WONCA ICPC-2 classification) and drug use;
- use of hypolipidaemic drug treatment (type of drug and dose);
- socio-demographic data, including age, sex, marital status, educational level and social class;
- beliefs and expectations about preventive recommendations: fulfilment of initial expectations in terms of improvement obtained and related drawbacks (questions with 5 Likert-type response options ranging from 1 “much more than expected” to 5 “much less than expected”); and,
- degree of satisfaction with the combined strategy according to a satisfaction questionnaire (Likert scale with 5 response options ranging from 1 “very dissatisfied” to 5 “very satisfied”).

### Statistical analysis

We will compare the variables of interest and the stratification and potentially confounding variables at baseline in both groups. The homogeneity of the two groups will be ascertained in terms of the baseline values of the study variables (Student’s t and chi-squared test, depending on the nature of the variables).

The subjects in the two groups will be classified into different LDL-C and total cholesterol reduction levels, and an initial crude analysis will be performed to assess the following parameters together with their respective 95% confidence intervals (CIs): absolute benefit increase (ABI); relative benefit increase (RBI); and number needed to treat (NNT).

Incidence of the outcome variables will be described and compared (proportion of subjects with appropriate control of lipid parameters and proportion of those who were guideline compliers) in both groups for each follow-up period (tests of comparison of proportions: Chi-squared test). Comparison of variations in lipid profiles in each group across the study will be made using the Student’s T-test or its non-parametric alternative (Mann–Whitney U test); and changes in the parameters in each group will be analysed using the T-test for repeated measures.

The possible existence of confounding factors or interaction of other variables in the relationship between the proposed intervention and the outcome variables will be assessed with the aid of logistic regression models (dependent variables: control of lipid parameters and therapeutic adherence). For continuous variables, assessment will be made using multiple linear regression.

Analysis will be conducted on an intention-to-treat basis, with all randomised patients being included in the analysis of effectiveness in accordance with their randomised group, regardless of the intervention received. In addition, a protocol analysis will also be performed for the main variables.

All analyses will be performed using the SPSS v20.0. computer software programme.

### Ethics approval

This study was approved by the Clinical Research Ethics Committee of the Albacete University Teaching Hospital on 27 March 2012. All participants signed the informed consent form to participate in the study.

## Discussion

Finding a type of action that could yield a higher success rate in the compliance and control of subjects with hypercholesterolaemia is fundamental when it comes to optimising available resources and improving the prevention and treatment of cardiovascular disease. This is why we feel that the most effective strategy will have to include activities targeted at enhancing knowledge about dyslipidaemia, reminding patients about guidelines and facilitating a higher patient participation rate. Similarly, we propose an action that is targeted, not only at pharmacological adherence, but also at the remaining recommendations for improving control of hypercholesterolaemia.

The results of the study will provide extremely useful information about the effectiveness of the proposed strategy of improving compliance in the prevention and management of cardiovascular disease based on increased control of lipid profile plasma levels. Should this intervention prove effective, a recommendation could then be issued on the application of the combined strategy to subjects with hypercholesterolaemia. Since it is a simple and relatively inexpensive intervention, it could be included as a recommendation in the respective clinical practice guidelines.

Selection biases will be reduced by using a randomisation procedure because it is not the researcher who allocates the intervention, and so any factor that might influence participation would affect both groups to the same degree. Likewise, confounding bias will be reduced by the effect of random chance, as a result of which any determinants that might influence the final result will tend to be shared between the intervention and control groups.

There is the possibility that the sample may not be representative of all eligible subjects, owing to differences between the persons who agree and those who do not agree to participate, posing a problem of external validity if the non-response rate proves to be high. For instance, persons who agree to participate in the study may possibly be more predisposed to follow guidelines than are the remaining candidates.

There could be a bias in data-collection due to the effect of regression towards the mean, applicable to analytical results. There is also the possibility of a risk of differential losses in follow-up between the groups compared, due to the duration of the study.

With the help of the health professionals who usually attend to the patients, every effort will be made to the reduce the number of losses to follow-up. Even so, in a case where such losses proved to be high and were not caused at random, a bias could appear in the measurements and compromise the study’s validity, due to the low representativeness of the sample.

The intervention proposal (combined strategy of improving adherence) does not allow for application of masking techniques, with only blind evaluation of results being feasible.

In view of the fact that the study is to be undertaken at various health centres in different Autonomous Regions, possible sources of error and their respective control mechanisms might be: protocol deviations (appropriate staff training will be undertaken); and data errors or omissions (these will be minimised using techniques for identifying anomalous data, and regular reports).

## Abbreviations

CDRFs: Cardiovascular disease risk factors
ABI: Absolute benefit increase
RBI: Relative benefit increase
NNT: Number needed to treat
CVR: Cardiovascular risk
TC: Total cholesterol
LDL-C: Low density lipoprotein cholesterol
HDL-C: High density lipoprotein cholesterol
SBP: Systolic blood pressure
DBP: Diastolic blood pressure.
